# Hours and State Control

**Published:** 1921-01-01

**Authors:** F. A. Sheldon

**Affiliations:** 14 St. Thomas's Street, London Bridge, S.E. 1


### Hours and State Control.

Sir,—I am glad to see that Miss Geraldine Bremner is asking for the opinion of all private nurses as to the desirability or otherwise of their hours of duty coming under State control, and Miss Seymour Yapp, in writing to the *Nursing Mirror*, most earnestly begs all College members to carefully consider and reply to the questionnaire recently issued to them. For seven years I have worked for private nurses, and I fully agree with Miss Bremner that they should be included in the Bill dealing with the employment and hours of nurses trained and in training.

Their special and most important branch of the profession could be safeguarded by the provision of a clause or order in which the interest of the patient and his home would be considered. A less arduous life would conserve the nurse's health and spirits, and would thus react favourably upon her patients, and, as Miss Seymour Yapp remarks, "there would still be opportunity for sacrifice." It would also lengthen her wage-earning life —a most important factor.

Will not some private nurses write suggesting schemes of hours and payment, and not merely contend, what we already know, that few patients could afford three nurses, and that nurses would refuse to go off duty at the eighth hour if treatment was due.

We must adapt the *form* of our profession (in all its branches) to changing times.

"Build thee more stately mansions

O my soul."

Not many generations ago it was pleaded that it was essential for the upkeep of working homes that children from the age of five should be employed in mills and mines. We shall see on January 1 a further reform, as "half-time" will come to an end, and no child under fourteen years may then be engaged in any industrial undertaking. It cannot be denied that these long-delayed ameliorations have brought, and will bring, benefits and not ruin in their train. Why should we dread change? It is Nature's law. "All that is humanely right is economically sound." Yours, etc.,

F. A. Sheldon.

14 St. Thomas's Street,
London Bridge, S.E. 1.
December 27, 1920.

